# Case report and literature review: Fabry disease misdiagnosing as polymyalgia rheumatica

**DOI:** 10.1097/MD.0000000000034630

**Published:** 2023-11-03

**Authors:** Wu Yanfang, He Juanjuan, Zhang Shengli, Yin Lei, Gao Fei

**Affiliations:** a Shengli Clinical Medical College of Fujian Medical University, Department of Rheumatology and Immunology of Fujian Provincial Hospital, Fuzhou, Fujian, China; b Shengli Clinical Medical College of Fujian Medical University, Department of Imaging of Fujian Provincial Hospital, Fuzhou, Fujian, China.

**Keywords:** Fabry disease, misdiagnosis, multiple organs, polymyalgia rheumatica, rare disease

## Abstract

**Rationale:**

The clinical manifestations of Fabry disease affect the nerves, kidneys, heart, skin, gastrointestinal tract and eyes. Our aim is to familiarize people with the FD diagnostic process by reporting this case.

**Patient concerns:**

A 79-year-old-male patient presented with muscle pain and weakness in the extremities, also with an increasing erythrocyte sedimentation rate and C-reactive protein. Further examinations revealed that multiple organ involvement, such as rash, myocardial hypertrophy, peripheral neuropathy.

**Diagnoses:**

Cardiac MR demonstrated hypertrophic cardiomyopathy, myocardial fibrosis and low myocardial T1 value. The patient was eventually diagnosed with Fabry disease through proteomics and genetic testing.

**Interventions:**

The treatment is enzyme replacement therapy (ERT). But this patient could not afford ERT and was given only general symptomatic treatment, pregabalin, and a gradual reduction in glucocorticoid.

**Outcomes:**

The patient’s symptoms of joint pain and muscle weakness reduced significantly, and ESR and CRP had decreased to normal.

**Lessons:**

FD is a rare disease and difficult to diagnose, but rare does not mean invisible. FD may present with signs and symptoms of rheumatic diseases. Rheumatologists should be aware and concerned about this disease.

## 1. Introduction

Fabry disease (FD) is a rare X-linked genetic lysosomal storage disorder caused by mutations in the α-galactosidase A (GLA) gene, resulting in deficiency of alpha-galactosidase A (α-Gal A) activity, leading to the progressive accumulation of glycosphingolipids, including the metabolic substrate globotriaosylceramide (GL-3) and globotriaosyl-sphingosine (Lyso-GL-3) in multiple organs. This causes a range of clinical manifestations and phenotypes or even potentially life-threatening complications. FD is charaterized by cardiac hypertrophy, kidney injury, central and peripheral nervous impairment, while rare to present with muscle disorder.^[1]^ Many physicians are still not similar to FD. We report a case of an old male patient presented with muscle pain and weakness, unexplained cardiac hypertrophy, and was finally diagnosed with FD by cardiac MRI and GLA gene sequence. By reporting this case, we hope to improve the understanding of FD among the rheumatologists and avoid delayed or incorrect diagnosis of FD.

## 2. Case description

### 2.1. Patient presentation

A 79-year-old Han male farmer was admitted to our department due to recurrent shoulder and lower limb muscle pain for 3 months unresponsive to non-steroidal anti-inflammatory drugs, accompanied by pain in elbow, knee and wrist joints, without joint swelling. The right shoulder joint magnetic resonance (MR): joint capsule, biceps longus tendon, sheath subacromial bursa and rostral subacromial bursa had a little fluid, erythrocyte sedimentation rate (ESR) 30 mm/hour, C-reactive protein (CRP) 20.5 mg/L. Then he was diagnosed with polymyalgia rheumatic (PMR) and given prednisone and hydroxychloroquine. The symptoms resolved quickly, ESR and CRP dropped to normal. The drug was stopped 2 weeks ago and the above symptoms reappeared and worsened before his current admission. The patient had a history of eczema for 3 years. He had no history of hypertension, diabetes, tumor or genetic disorders.

### 2.2. Physical examinations

The patient had scattered petechiae on both lower limbs and anterior chest and tongue hypertrophy. The heart borders was enlarged to the left, and rate and rhythm were normal. The shoulder joints were limited in abduction, Joints tenderness of shoulder, elbow, knee and wrist were positive, without swelling. The muscle strength and tone of the extremities are normal, the sensory symmetry of the lower extremities is diminished.

### 2.3. Diagnosis process

**Figure 1.**
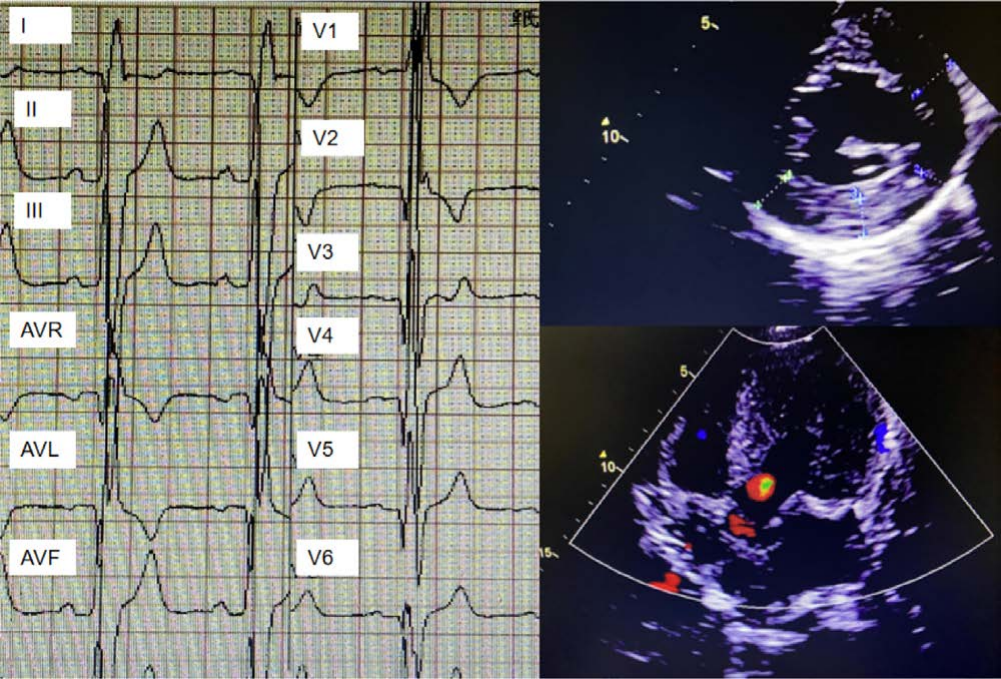
The patient presented with significant cardiac hypertrophy (A) Electrocardiography showed High QRS voltages and giant negative T-waves. (B) Doppler ultronosography confirmed septal and left ventricular myocardial hypertrophy.

**Figure 2.**
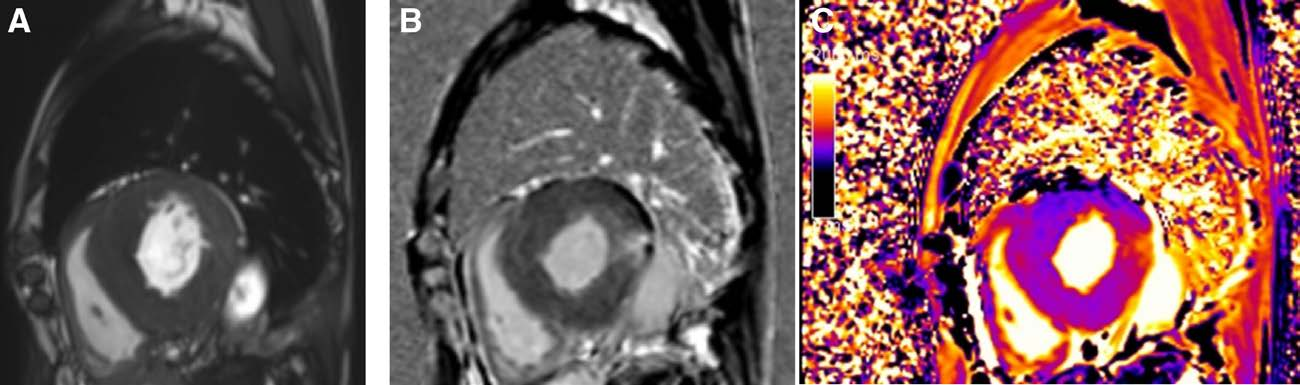
Cardiac magnetic resonance revealed classic images of Fabry disease (FD) (A) Basal segment hypertrophic cardiomyopathy (HCM), (B) Basal inferolateral late gadolinium enhancement (LGE) with fibrosis, (C) Low myocardial T1 values appearing purple.

**Figure 3.**
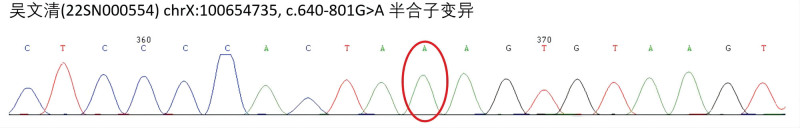
Gene sequencing identified GLA gene mutation located in the chrX:100654735,c.640-801G > A. GLA = α-galactosidase A.

After hospitalization, the patient completed relevant tests. ESR 90 mm/hour, CRP 155 mg/L, rheumatoid factor 46.3 IU/mL; HLA-B27 positive; autoimmune antibodies including anti-nuclear antibody, anti-neutrophil cytoplasmic antibody and CCP were negative. Tumor markers were all normal. Troponin I was 0.26 ng/mL; NT-pro-BNP was 1702 Pg/mL; electrocardiogram (Fig. 1A) indicated abnormal Q waves, complete right bundle branch block and left anterior branch block, high ventricular voltage, bidirectional and deep inversion of T waves. Cardiac ultrasound (Fig. 1B) showed septum and left ventricular myocardial hypertrophy (septum 1.59 cm, left ventricular 1.33 cm), reduced left ventricular diastolic function and pericardial effusion (trace). Electromyography revealed reduced motor wave amplitude of the ulnar nerve, common peroneal nerve and right tibial nerve bilaterally and prolonged F-response latency of the right median nerve and bilateral tibial nerves. X-ray, computed tomography, and MR of sacroiliac joint did not indicate bone marrow edema or joint space narrowing. Then, cardiac MR demonstrated hypertrophic cardiomyopathy (Fig. 2A), myocardial fibrosis (Fig. 2B) and low myocardial T1 value (Fig. 2C). These images revealed FD. Finally, α-Gal A activity, Lyso-GL-3 and GLA gene sequence were detected. Lyso-GL-3 4.78 ng/mL (reference value: <1.11 ng/mL) and GLA gene mutation (Fig. 3) located in the chrX:100654735,c.640-801G > A confirmed the diagnosis: FD.

## 3. Discussion

FD is a rare disease with a predicted prevalence of 1/117,000 to 1/40,000 in the general population.^[2,3]^ It was first reported by Fabry et al in 1898 and is an X-linked recessive disorder in which a mutation in the GLA gene caused deficiency of α-Gal A activity and consequent accumulation of glycosphingolipids mainly GL-3 and lyso-GL-3 in body fluids and lysosomes of the cells. Abnormal GL-3 and lyso-GL-3 leaded to multi-system damage.^[4]^

FD exhibits many phenotypes, ranging from severe early onset to mild or atypical late onset, and is associated with over 1000 genotypes.^[5]^ Patients often have a variety of clinical signs and symptoms, mostly chronic or intermittent burning pain and sensory abnormalities in the extremities, impaired sweating (mostly manifesting as little or no sweating); cutaneous angiokeratomas in the lumbar region; radiolucent deposits visible under the corneal slit lamp, hearing loss and vestibular dysfunction; cardiac hypertrophy, cardiac conduction abnormalities, cardiac valve disease and heart failure; cerebrovascular and other organ involvement. The diagnosis of FD is based on one of the following criteria according Chinese FD Expert Panel,^[6]^ GLA gene mutation, decreased α-Gal A enzyme activity, increased plasma Lyso-GL-3 levels, pathological examination of renal, skin, myocardial and neurological tissues with corresponding histoplasmic vacuolar changes on light microscopy and intracytoplasmic filling with starved “myeloid vesicles” on electron microscopy. Our patient was a male with a late onset of the disease and had significant cardiac lesions, gastrointestinal symptoms and extremity sensory abnormalities and the diagnosis was finally clarified based on his plasma Lyso-GL-3 level and GLA gene mutation. But his α-Gal A activity was not reduced. We therefore reviewed the literature. Plasma Lyso-GL-3, a cationic amphiphilic molecule containing an acute glycosyl group and relatively hydrophilic, had recently been emphasized by several domestic and international guidelines and consensus on the importance of its role in the diagnosis, screening, and monitoring of FD, as one of the biomarkers specific for FD.^[7,8]^ Maruyama et al^[9]^ subjected 3 Japanese women with significantly elevated levels of Lyso-GL-3, although conventional genetic testing did not reveal major GLA mutations associated with FD, intron analysis revealed mutations of unknown significance, and the 3 cases were finally diagnosed with FD by further histopathological examination of the biopsies. Another relevant literature indicated^[10]^ that the sensitivity of plasma LysoGL3 for the diagnosis of FD in women was higher than that of α-Gal A activity assay (82.4% vs 23.5%). Therefore, we believe that high levels of Lyso-GL-3 are more important for confirming the diagnosis of FD.

Patients may seek consultation in rheumatology for musculoskeletal disorders, or combined with the multi-systemic damage, and misdiagnosed as rheumatic diseases such as undifferentiated arthritis and systemic vasculitis. In a retrospective study,^[11,12]^ 107 adult patients with confirmed FD, 70 men and 37 women aged 18 to 69 years (median 37 years), 28 patients (26.2%) had at least one referral to a rheumatologist prior to diagnosis, with possible causes including “true” arthralgia (n = 8), unexplained fever (n = 11), raynaud phenomenon (n = 2) and laboratory markers of inflammation (n = 12) were misdiagnosed as rheumatic diseases. These patients were treated with glucocorticoids and immunosuppressive agents and even biological therapies. Moreover, Vordenbäumen et al showed that approximately 10% of patients with FD presented with “genuine” joint pain, episodes of unexplained fever and/or elevated markers of inflammation, that is, ESR and CRP, which were usually regarded by physicians as indicators of rheumatic disease.^[12]^ Additionally a significant delay of FD diagnosis from first symptoms was often reported.^[13,14]^ Joint discomfort is also a common clinical feature of FD.^[15]^ Our patient also consulted the rheumatology department because of joint and muscle pain, high ESR and CRP. According to the diagnostic criteria of rheumatic polymyalgia, the patient did fit the PMR, but the final diagnosis of FD was made because of the combination of unexplained cardiac hypertrophy and myocardial injury. The above evidence raised concerns that FD may be overlooked in rheumatological practice.

When FD has hypertrophy as its main manifestation, it may be clinically grouped with hypertrophic cardiomyopathy prior to genetic testing. Wei-Xian Yang^[16]^et al reported that FD is not rare in hypertrophic cardiomyopathy patients in China, with an incidence of 0.93%. T1 mapping of cardiac MR can potentially detect early cardiac damage and differentiate left ventricular hypertrophy (LVH) due to FD from other diseases.^[17]^ In an resent research,^[18]^ cardiac MR was performed on 227 study subjects including patients with FD, healthy volunteers and patients with other diseases causing LVH (including hypertension, hypertrophic cardiomyopathy, severe aortic stenosis and cardiac amyloidosis), and it was found that ventricular septal T1 was lower in patients with Fabray disease compared with healthy volunteers, and T1 was negatively correlated with cardiac wall thickness (*r* = −0.51; *P* = .0004), whereas patients with other diseases had higher septal T1, suggesting that T1 noncontrast imaging by cardiac MRI has unique and powerful measurement potential in the imaging evaluation of LVH and FD. In this case, the patient was also found low T1 value and LVH on cardiac MR, and recommended to exclude FD. Finally he was later confirmed FD by blood α-galactosidase A enzyme activity, plasma Lyso-GL-3 level and genetic testing.

The treatment is divided into general symptomatic treatment and enzyme replacement therapy (ERT).^[19]^ ERT can relieve some symptoms of FD. Unfortunately this patient could not afford ERT and was given only pregabalin and a gradual reduction in glucocorticoid, the patient symptoms of joint pain and muscle weakness improved significantly, and ESR and CRP had decreased to normal, but cardiac MR had not reviewed.

When we found that the patient myocardial hypertrophy and peripheral nerve injury could not be explained by PMR, we performed cardiac MR and genetic tests, which finally enabled the diagnosis of rare diseases and avoided misdiagnosis. Our sensitivity to rare diseases is a point of pride. The disadvantage is that no further family lineage screening was performed. Because the patient mother and aunt were both deceased and he did not have a daughter.

## 4. Conclusions

In summary, FD is a rare disease and difficult to diagnose, but rare does not mean invisible. FD may present with signs and symptoms of rheumatic diseases. Patients may seek clinical advice from the rheumatology department for pain in the limbs, and clinical rheumatologists should be aware and concerned about this disease. By screening high-risk groups, the diagnosis rate of FD can be improved. Quality of life and prognosis of patients can be improved through effective measures

## Author contributions

**Conceptualization:** Yanfang Wu, Fei Gao.

**Data curation:** Yanfang Wu, Juanjuan He.

**Investigation:** Shengli Zhang.

**Supervision:** Fei Gao, Shengli Zhang.

**Validation:** Juanjuan He, Shengli Zhang.

**Visualization:** Lei Yin.

**Writing – original draft:** Yanfang Wu.

**Writing – review & editing:** Fei Gao.

## References

[1] Ortiz A Germain DP Desnick RJ. Fabry disease revisited: management and treatment recommendations for adult patients. Mol Genet Metab. 2018;123:416–27. 29530533 10.1016/j.ymgme.2018.02.014

[2] Evans WR Rafi I. Rare diseases in general practice: recognising the Zebras among the horses. Br J Gen Pract. 2016;66:550–1. 27789486 10.3399/bjgp16X687625 PMC5072891

[3] Mehta A Beck M Eyskens F. Fabry disease: a review of current management strategies. QJM. 2010;103:641–59. 20660166 10.1093/qjmed/hcq117

[4] Li X Ren X Zhang Y. Fabry disease: mechanism and therapeutics strategies. Front Pharmacol. 2022;13:1025740. 36386210 10.3389/fphar.2022.1025740 PMC9643830

[5] Zarate YA Hopkin RJ. Fabry’s disease. Lancet. 2008;372:1427–35. 18940466 10.1016/S0140-6736(08)61589-5

[6] Chinese Fabry Disease Expert Panel. Expert consensus for diagnosis and treatment of Fabry disease in China (2021). Zhonghua Nei Ke Za Zhi. 2021;60:321–30. 33765701 10.3760/cma.j.cn112138-20201218-01028

[7] Lukas J Giese AK Markoff A. Functional characterisation of alpha-galactosidase a mutations as a basis for a new classification system in fabry disease. PLoS Genet. 2013;9:e1003632. 23935525 10.1371/journal.pgen.1003632 PMC3731228

[8] Sakuraba H Togawa T Tsukimura T. Plasma lyso-Gb3: a biomarker for monitoring Fabry patients during enzyme replacement therapy. Clin Exp Nephrol. 2018;22:843–9. 29288396 10.1007/s10157-017-1525-3

[9] Maruyama H Miyata K Mikame M. Effectiveness of plasma lyso-Gb3 as a biomarker for selecting high-risk patients with Fabry disease from multispecialty clinics for genetic analysis. Genet Med. 2019;21:44–52. 29543226 10.1038/gim.2018.31 PMC6363642

[10] Ouyang Y Chen B Pan X. Clinical significance of plasma globotriaosylsphingosine levels in Chinese patients with Fabry disease. Exp Ther Med. 2018;15:3733–42. 29563981 10.3892/etm.2018.5889 PMC5858121

[11] Moiseev S Karovaikina E Novikov PI. What rheumatologist should know about Fabry disease. Ann Rheum Dis. 2020;79:e71. 31040120 10.1136/annrheumdis-2019-215476

[12] Vordenbäumen S Brinks R Richter JG. Clinical characteristics of patients with alpha-galactosidase A gene variants in a German multicentre cohort of early undifferentiated arthritis. Ann Rheum Dis. 2019;78:1286–7. 30902821 10.1136/annrheumdis-2019-215223

[13] Lidove O Zeller V Chicheportiche V. Musculoskeletal manifestations of fabry disease: a retrospective study. Joint Bone Spine. 2016;83:421–6. 26697993 10.1016/j.jbspin.2015.11.001

[14] Lidove O Barbey F Niu DM. Fabry in the older patient: clinical consequences and possibilities for treatment. Mol Genet Metab. 2016;118:319–25. 27221354 10.1016/j.ymgme.2016.05.009

[15] Ivleva A Weith E Mehta A. The influence of patient-reported joint manifestations on quality of life in Fabry patients. JIMD Rep. 2018;41:37–45. 29380258 10.1007/8904_2017_84 PMC6122052

[16] Xiao Y Sun Y Tian T. Prevalence and clinical characteristics of Fabry disease in Chinese patients with hypertrophic cardiomyopathy. Am J Med Sci. 2021;362:260–7. 34266644 10.1016/j.amjms.2021.01.009

[17] Karamitsos TD Piechnik SK Banypersad SM. Noncontrast T1 mapping for the diagnosis of cardiac amyloidosis. JACC Cardiovasc Imaging. 2013;6:488–97. 23498672 10.1016/j.jcmg.2012.11.013

[18] Sado DM White SK Piechnik SK. Identification and assessment of Anderson-Fabry disease by cardiovascular magnetic resonance noncontrast myocardial T1 mapping. Circ Cardiovasc Imaging. 2013;6:392–8. 23564562 10.1161/CIRCIMAGING.112.000070

[19] Lenders M Brand E. Fabry disease: the current treatment landscape. Drugs. 2021;81:635–45. 33721270 10.1007/s40265-021-01486-1 PMC8102455

